# Cytotoxic lymphocyte effector function is unaffected in patients with Gaucher disease

**DOI:** 10.3389/fimmu.2025.1680520

**Published:** 2025-10-16

**Authors:** Jinyun Zou, Tahereh Noori, Mark Walterfang, Jeff Szer, Joseph A. Trapani, Ilia Voskoboinik

**Affiliations:** ^1^ Killer Cell Biology Laboratory, Cancer Immunology Program, Peter MacCallum Cancer Centre, Melbourne, VIC, Australia; ^2^ Sir Peter MacCallum Department of Oncology, University of Melbourne, Parkville, VIC, Australia; ^3^ Oxidation Biology Unit, Melbourne Dementia Research Centre, Florey Institute of Neuroscience and Mental Health and The University of Melbourne, Parkville, VIC, Australia; ^4^ Neuropsychiatry Unit, Royal Melbourne Hospital, Melbourne, VIC, Australia; ^5^ Melbourne Neuropsychiatry Centre, University of Melbourne, Parkville, VIC, Australia; ^6^ Clinical Hematology, Peter MacCallum Cancer Centre and Royal Melbourne Hospital, Melbourne, VIC, Australia; ^7^ Department of Medicine, The University of Melbourne, Parkville, VIC, Australia; ^8^ Cancer Cell Death Laboratory, Cancer Immunology Program, Peter MacCallum Cancer Centre, Melbourne, VIC, Australia

**Keywords:** lysosomal storage disorders, CD8+ T cells, natural killer cells, hemophagocytic lymphohistiocytosis, sphingolipidosis

## Abstract

Gaucher disease (GD) is one of the most common lysosomal storage disorders. It is caused by bi-allelic mutations in the *GBA1* gene responsible for the production of β-glucocerebrosidase, an enzyme responsible for the hydrolysis of the sphingolipid glucocerebroside. This results in its accumulation in various organs, and patients can present with a variety of symptoms ranging from visceral enlargement, bone pathology and hematological manifestations. Neuronopathic effects are seen in the severe form of the disease. GD patients also have an increased risk of B-cell malignancies. Some of the hematological symptoms of GD resemble those of the systemic hyperinflammatory condition, hemophagocytic lymphohistiocytosis (HLH). HLH can be familial, due to functional deficiencies in cytotoxic lymphocytes, or acquired from a variety of causes ranging from infections to blood cancers. While patients with inherited and acquired HLH receive the same first-line therapy, patients with the familial form can only be cured by stem cell transplantation, although this treatment may be detrimental to patients with the acquired form of the disease. Therefore, we investigated whether the abnormal lipid accumulation in GD patient cytotoxic lymphocytes and in cells with irreversibly inhibited glucocerebrosidase activity affects their cytotoxicity. Our detailed analysis of primary cytotoxic T lymphocytes and natural killer cells revealed that the activity of these cells was not affected. This finding has important implications for the treatment choices for patients with GD and suggests that these patients can be treated with autologous immunotherapy if they develop hematological cancers.

## Introduction

Gaucher disease (GD) is one of the most common lysosomal storage disorders (LSDs) and is caused by mutations in the *GBA1* gene (OMIM# 606463), which encodes the lysosomal enzyme, β-glucocerebrosidase (GCase). The deficiency of GCase results in the accumulation of sphingolipids (primarily glucocerebroside), within lysosomes (1, 2). GD has been categorized into 3 subtypes based on clinical presentation (3, 4). GD Type I (non-neuronopathic form) is the most frequent subtype and may present in childhood or later in life with hepatosplenomegaly, cytopenia and bone marrow infiltration by characteristic macrophages, known as “Gaucher cells.” GD Type II (acute neuronopathic form) is a rare and severe form of the disease with early onset. It is usually diagnosed in infants between a few months and one year of age and presents with acute neurodegeneration, leading to death within the first two years of life. GD Type III (subacute neuronopathic form) is characterized by abnormal eye movements, epilepsy, and dementia resulting from chronic neuroinflammation. The severity and the incidence of GD Type III are intermediate between those of Types I and II.

To date, several studies have demonstrated that the immune system in GD patients is significantly altered. Thus, macrophage infiltration is observed in the liver, spleen, and bone marrow, contributing to hepatosplenomegaly, cytopenia, and lytic bone lesions. In addition, GD is associated with chronic inflammation, characterized by elevated levels of polyclonal immunoglobulins (IgA, IgG, and IgM) (5), chemokines (CCL2, CXCL1, and CXCL8), and upregulated expression of pro-inflammatory cytokines such as IFN-γ, TNF-α, IL-1β, and IL-6 (6). Finally, GD shows a strong association with B-cell malignancies (7–9).

Hepatosplenomegaly, cytopenia, macrophage infiltration, and chronic immune activation are also hallmark features of both acquired and familial hemophagocytic lymphohistiocytosis (HLH) - immune disorders characterized by uncontrolled immune activation that leads to severe systemic inflammation. Familial HLH (FHL) can affect individuals of any age, from infancy to adulthood. The disease is caused by impaired effector function of cytotoxic lymphocytes, including cytotoxic T lymphocytes (CTLs) and natural killer (NK) cells, due to mutations that disrupt the production or secretion of the pore-forming effector protein perforin—a condition also referred to as “perforinopathy” (10). Importantly, partial cytotoxic lymphocyte deficiency caused by hypomorphic mutations in FHL-associated genes (11, 12) often results in an atypical, late-onset form of FHL that may be difficult to diagnose due to its cryptic and variable clinical presentation (13, 14).

Several clinical cases of HLH have been reported in association with GD, and overlapping symptoms between the two conditions can further complicate diagnosis in clinical practice (15–19). As in GD, incomplete loss of cytotoxic lymphocyte effector function due to hypomorphic mutations in FHL-associated genes has also been linked to non-Hodgkin lymphoma, suggesting a shared role in immune surveillance of hematological cancers. Moreover, the pore-forming activity of perforin has been shown to depend on the phospholipid environment. In another sphingolipidosis, Niemann-Pick Disease type C1, the accumulation of sphingomyelin and cholesterol within cytotoxic granules severely impairs perforin activity and cytotoxic lymphocyte function (20). Despite the similarities in the systemic clinical manifestations of GD and FHL, the impact of GCase deficiency and impaired sphingolipid metabolism, in general, on cytotoxic lymphocyte function remains unknown.

Here, we comprehensively investigated the effector functions of cytotoxic T lymphocytes and natural killer cells in GD patients and in GCase-deficient CTL/NK cells and found no functional defect in either cell type.

## Methods

### Patients

Blood samples from five GD Type I patients and four adult healthy donors (HDs) were collected, and peripheral blood mononuclear cells (PBMCs) were isolated using Ficoll^R^ Paque Plus (GE Healthcare, Illinois, USA) following a standard protocol (14). All patients received enzyme replacement therapy once every two weeks (**Table 1**), and blood samples were collected immediately before drug administration to minimize any treatment effects on the cells.

**Table 1:** Patient information and treatment details.

Patient	Age till now	Mutation	Age of diagnosis	ERT dosage	Healthy donor
Patient #1	66	c.475C>T (p.Arg159Trp) Het	47	Taliglucerase 2200U Q2W (30U/kg)	Healthy Donor 1
Patient #2	66	c.1226A>G hom (N370S)	59	Velaglucerase 7200U Q2W (60U/kg)	
Patient #3	56	N370S/IVS2 + 1	44	Velaglucerase 2800U Q2W (30U/kg)	Healthy Donor 3
Patient #4	60	c.764T>A (p.Phe255Tyr) Het	42	Velaglucerase 5600U Q2W (60U/kg)	Healthy Donor 4
Patient #5	58	c.1226A>G hom (N370S)	40	Velaglucerase 1200U Q2W (20U/kg)	Healthy Donor 5

ERT, enzyme replacement therapy, once every two weeks.

### Cell lines

P815 and K562 cell lines were cultured in RPMI-1640 medium supplemented with 10% fetal bovine serum (Cytiva, Massachusetts, USA) and 2 mM GlutaMAX (Gibco, Cat# 35050061) at 37°C, 5% CO_2_. PBMCs or isolated CD8+ T cells (CTLs) were maintained in RPMI-1640 media supplemented with 10% fetal bovine serum, 2 mM GlutaMAX, 50 IU/Penicillin-Streptomycin, 1 mM HEPES (Gibco, Cat# 15630080), 100 µM non-essential amino acids (Gibco, Cat#11140050), 1 mM sodium pyruvate (Gibco, Cat# 11360070), 50 µM 2-mercaptoethanol (Sigma-Aldrich, St. Louis, USA). Cells were cultured in media supplemented with rhIL-2 at a different concentration as described in the following section. KHYG1 cells were cultured in the same media supplemented with 450 IU/mL of rhIL-2.

### CTL and natural killer cell culture

Freshly isolated PBMCs were used to set up CTL and NK cell functional assays. For CTL, PBMCs were resuspended in CTL media at 5 x 10^5^ cells/mL supplemented with 100 IU/mL of rhIL-2. CTLs were activated by adding OKT3 mAb (Cat#. 16-0037-85, Thermo Fisher Scientific, USA) at a final concentration of 30 ng/mL, and cells were seeded in 24-well plates. On day 3, cells were resuspended in fresh media supplemented with 100 IU/mL of rhIL-2 and CTLs (CD4-, CD16-, CD8+) were isolated on day 7 using a Fluorescence-activated cell sorter. CTL functional assays were performed on day 10.

For NK cell functional assays, freshly isolated PBMCs were resuspended in CTL media supplemented with or without rhIL-2 (100 IU/mL). Resuspended cells were cultured in 24-well plates overnight, at 10^6^ cells/mL. Effector (NK)-to-target cell ratios were standardized according to the percentage of cytotoxic NK cells in PBMCs (CD3-, CD16+, CD56dim).

### ^51^Cr-release assay

Target cells - K562 cells for NK cells or P815 cells for CTL - were labelled with 100 µCi of Na_2_^51^CrO_4_ for one hour at 37°C, 5% CO_2_. CTL cytotoxicity was assessed by performing 4-hour reverse antigen-dependent cellular cytotoxicity (ADCC) assays, utilizing Fcγ receptors on P815 target cells to bind the Fc portion of anti-CD3 mAb (OKT3, 1 µg/mL) directed against the T cells. NK cytotoxicity was assessed by co-culturing PBMCs with K562 for 4 hours. The number of NK cells was estimated by normalizing to their percentage (CD3-, CD16+, CD56dim) within the PBMCs population. Details were described in a previous study (14).

### Degranulation assay

Degranulation assay was performed by co-culturing effector cells and target cells in a 96-well U-bottom plate at 37°C, 5% CO_2_, for 4 hours, in the presence of anti-human LAMP1 (CD107a) antibody (Cat# 328610, BioLegend,1:50 dilution). Isolated CTLs were co-cultured with P815 cells and OKT3 mAb as described above. After incubation, CTLs were labelled with anti-CD8 (Cat#. 344748, BioLegend) for 15 minutes. The level of CTL degranulation was assessed by comparing the portion of LAMP1-positive cells incubated in the presence or absence of OKT3. For NK cell degranulation assay, PBMCs were co-cultured with K562 cells. NK cells were then labelled with anti-CD3 (Cat#. 560176, BD Bioscience), anti-CD16 (Cat#. 302044, BioLegend), and anti-CD56 (Cat#. 362508, BioLegend), for 15 minutes. Cells were analyzed using the BD LSR II flow cytometer. Biological replicates are specified in the figure legend. The cytotoxic NK cell population in PBMCs was identified by applying (CD3-, CD16+, CD56dim) on flow cytometry. The level of NK degranulation was assessed by comparing the LAMP1-positive population in the presence or absence of target cells.

### Glucocerebrosidase enzymatic assay

Cells were lysed in ice-cold non-denaturing lysis buffer (1% Triton X-100, 150 mM NaCl, 50 mM Tri-HCl, pH 8.5) containing cOmplete ULTRA protease inhibitor cocktail (Cat#. 05892970001, Roche, Switzerland) for 30 minutes on ice, and centrifuged for 10 minutes at 16,000xg at 4°C, to remove cell debris and nuclei. Total protein concentration was quantified using the BCA assay (Cat#.23225 Thermo Fisher Scientific). 40 µg of extracted protein were mixed with activity assay buffer (Citrate-Phosphate buffer pH 5.4, 10 mM Sodium Taurocholate, 1 mM EDTA). 4-Methylumbelliferyl-β-D-Glucopyranoside (Cat#. 20948, Cayman Chemical, USA) was added to the reaction at a final concentration of 1 mM, and the mixture was incubated at 37°C for 1 hour. Following incubation, the reaction was terminated by adding an equal volume of stopping buffer (1 M Glycine-NaOH buffer, pH 10.6). Fluorescence intensity was measured by the Citation 3 Plate Reader (BioTek) with excitation/emission wavelength of 360nm/450nm. Fluorescence readings were normalized to the blank (no cell lysate) group.

Conduritol β-epoxide, CBE (Cat#. 15216, Cayman Chemical), is a specific and irreversible inhibitor of GCase (21). It was dissolved in sterile PBS at 10 mM and stored at -20°C. Experimental cells were resuspended in culture media supplemented with 100 µM CBE for 7 and 14 days, and the media was replaced every two days with fresh media containing 100 µM CBE.

### Statistical analysis

Statistical analysis was performed using a Mann-Whitney U-test (Prism v10.2.3).

### Ethics

This study was approved by the Royal Melbourne Hospital Human Research Ethics Committee HREC/64887/MH.

## Results

### GD patient-derived CTL and NK cells exhibit normal cytotoxic activity

**Figure 1:**
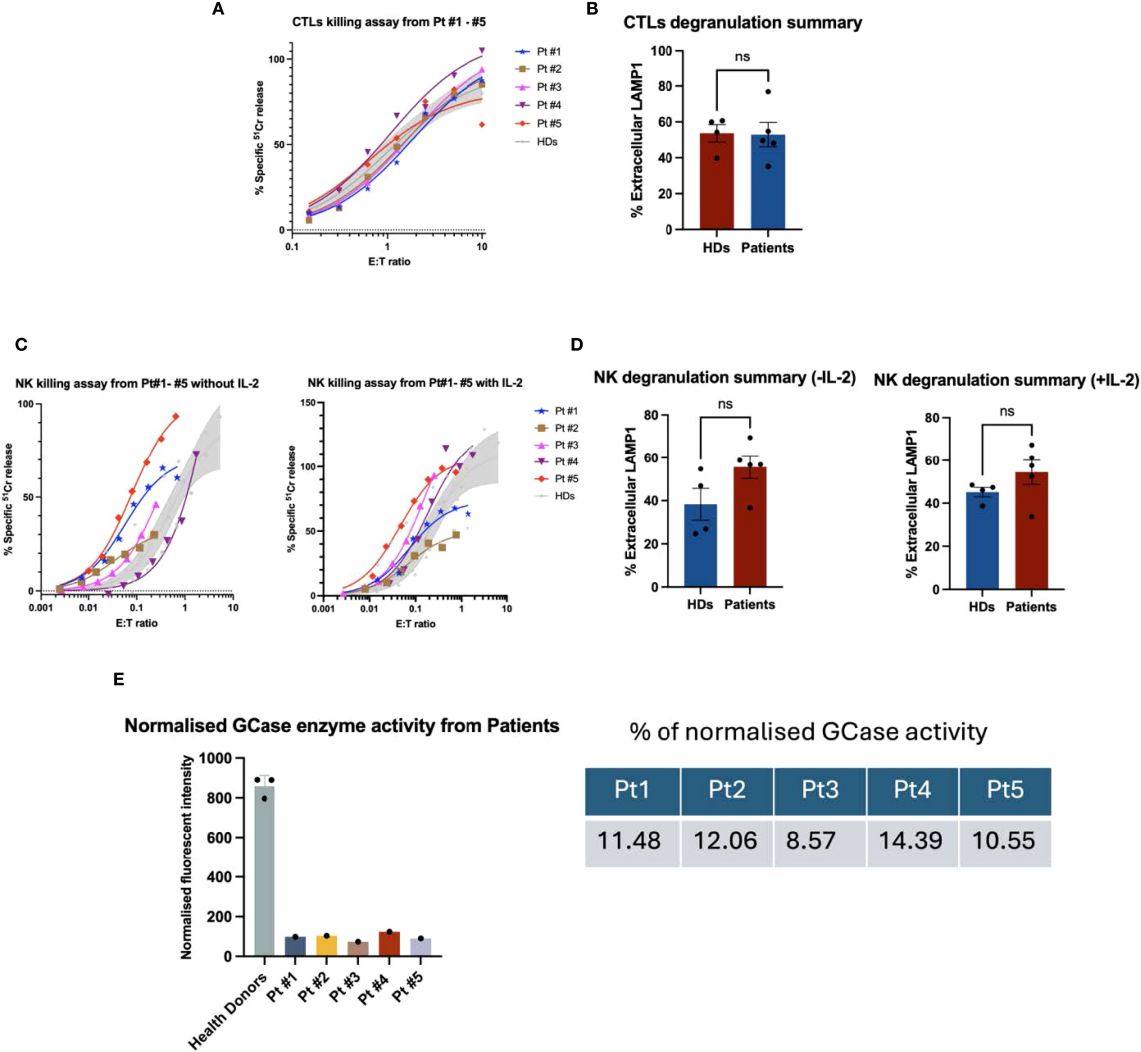
Functional analysis of GD patient-derived CTL cells and NK cells. **(A)** ^51^Cr release assay performed at different effector-to-target (E:T) ratios showed that cytotoxic T lymphocytes (CTLs) from all five GD patients exhibited cytotoxic activity comparable to that of healthy donors. The shaded area represents the mean cytotoxicity of healthy donors (*n* = 4), with a 95% confidence interval. **(B)** Summary results of the CTL degranulation assay are presented as mean ± SEM. LAMP1 (CD107a) expression was assessed by co-culturing effector and target cells in the presence or absence of OKT3. *n* = 4 for healthy donors and *n* = 5 for GD patients. The percentage of extracellular LAMP1 was calculated as the difference in the proportion of LAMP1-positive cells incubated with P815 targets in the absence and in the presence of OKT3. CTL degranulation in GD patients was not significantly different from that of healthy donors (“ns”). The data were analysed using the Mann-Whitney U test. **(C)** Peripheral blood mononuclear cells (PBMCs) from patients and healthy donors were cultured overnight in the absence or presence of recombinant human IL-2 (rhIL-2, 100 IU/mL). Natural killer (NK) cell cytotoxicity was assessed against K562 target cells using a ^51^Cr release assay. The effector-to-target (E: T) ratio was standardised based on the percentage of NK cells within the PBMC population, defined as CD3^-^/CD16^+^/CD56^dim^. The shaded area represents the mean cytotoxicity of healthy donors (*n* = 4), with a 95% confidence interval. Patient-derived NK cells exhibited cytotoxic activity comparable to that of healthy donors, both in the absence and presence of rhIL-2. **(D)** In the NK cell degranulation assay, the percentage of extracellular LAMP1 (CD107a) was calculated as the difference in the proportion of LAMP1-positive NK cells incubated with and without K562 target cells. NK cells from GD patients exhibited normal levels of degranulation compared to those from healthy donors. Shown is mean ± SEM (*n* = 4 and 5, for healthy donors and GD patients, respectively). NK degranulation in GD patients was not significantly different from that of healthy donors (“ns”). The data were analysed using the Mann-Whitney U test. **(E)** GCase enzyme activity in patient-derived cytotoxic T lymphocytes (CTLs) was assessed by incubating cell lysates with a GCase-specific substrate, 4-methylumbelliferyl-β-D-glucopyranoside, for one hour. Following incubation, fluorescence intensity was measured using a plate reader at excitation/emission wavelengths of 360/450 nm. Fluorescence values were normalized by subtracting the mean of the blank (substrate only), and then dividing by the difference between the mean fluorescence of healthy donor (HD) controls and the blank. As shown in the figure, all five patients exhibited low GCase enzyme activity, ranging from 8.6% to 14.4% compared to HD controls (*n* = 3 biological replicates).

To investigate the cytotoxic activity of CTL/NK cells, we isolated PBMCs from GD Type I patients (**Table 1**). Blood from control healthy donors was collected at the same time. All patients were receiving enzyme replacement therapy (ERT) fortnightly, and blood samples were taken immediately before treatment. CTLs (CD8+ T cells) were activated using OKT3, isolated by FACS, and tested for activity using a redirected (‘reverse’) cytotoxicity assay against P815 cells in the presence of OKT3 (anti-CD3 antibody). No differences in cytotoxicity or degranulation were observed between CTL cells from GD patients and healthy donors (**Figures 1A, B**). Similarly, NK cells from all five patients exhibited normal cytotoxicity and degranulation capacity against K562 target cells both in the absence and presence of rhIL-2, compared with NK cell function in healthy donors (**Figures 1C, D**).

### Reduced GCase activity in patient cytotoxic T lymphocytes

Considering the relatively mild symptoms of GD type I patients, we predicted that mutant GCase may retain some residual enzymatic activity. Indeed, at least one mutation, N370S, has previously been shown to result in protein misfolding without complete loss of function (22). In addition, patients had been receiving ERT for several years, which may have also contributed to GCase activity. Taking these factors together, we hypothesized that patient CTL/NK cells might retain some GCase activity, and we therefore measured it in CTLs using the enzyme-specific fluorometric substrate 4-methylumbelliferyl ß-D-glucuronide. Enzymatic assays revealed severely reduced, but not absent, GCase activity in patient CTLs: 8–14% of the level observed in healthy donors (**Figure 1E**). This level of activity was consistent with GD type 1, where residual GCase function accounts for the mild neurological symptoms (23). This residual activity may also help explain the preserved CTL and NK function in patients. To explore this possibility further, and to rule out other factors that could affect cytotoxic lymphocyte activity, we assessed the effect of the specific GCase inhibitor, conduritol β-epoxide (CBE), on CTL/NK cell function.

### Inhibition of GCase does not affect CTL and NK cell cytotoxicity

**Figure 2:**
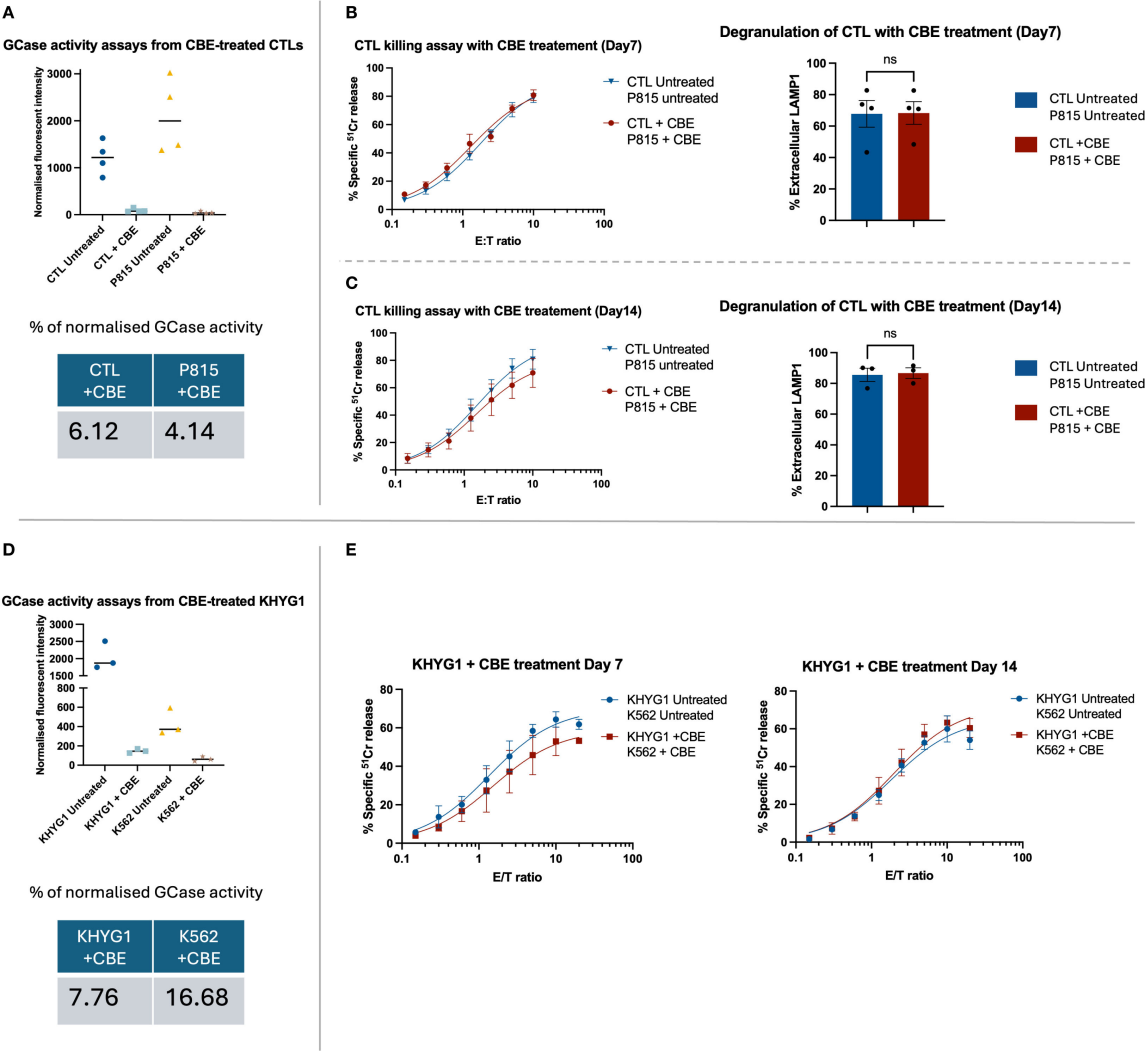
The functional assays for primary CTLs and a NK cell line (KHYG1) treated with GCase inhibitor, CBE. **(A, D)** The enzymatic assay confirmed effective suppression of GCase activity in both CTL and KHYG1 cells. Fluorescence intensity was normalised by subtracting the mean of the blank (substrate only) and then dividing by the difference between the mean fluorescence of untreated samples and the blank. Although the activity of GCase was successfully inhibited in K562 treated with CBE, the overall percentage of enzyme activity did not appear markedly reduced due to the cell line’s inherently low basal expression of GCase. **(B, C)** The healthy donor CTLs and target P815 cells were treated with 100 µM of CBE for up to 14 days, with fresh inhibitor added every second day. Functional assays were performed on days 7 and 14 of CBE treatment. CTL cytotoxicity and degranulation were not affected by the CBE treatment. Data are presented as mean ± SEM; *n* = 3 or 4 biological replicates, as shown in the figures. “ns” indicates no significant difference. *n* = 4 **(B)** and *n* = 3 **(C)** independent experiments. The data were analysed using the Mann-Whitney U test. **(E)** The KHYG1 and K562 were treated with 100 µM CBE for up to 14 days. Cytotoxicity assays were performed on days 7 and 14 of CBE treatment. The killing capacity of KHYG1 cells was not significantly affected by CBE. Shown is the mean ± SEM of three independent experiments.

To assess the effect of GCase inhibition on CTL activity, healthy donor cells and target P815 cells were cultured in the presence of 100 µM CBE to inhibit GCase activity in both cell types. This approach better reflects *in vivo* conditions, in which both effector and target cells in patients are GCase-deficient. After 7 days of treatment, GCase activity was reduced by at least 93% in both cell types compared to the untreated group (**Figure 2A**). Functional assays (**Figures 2B, C**) demonstrated unchanged cytotoxicity and degranulation in both CBE-treated and untreated CTLs on day 7 and day 14 (**Supplementary Figure S3**).

To test the effect of GCase inhibition on NK cells, we used KHYG1 cell line, which is commonly employed to study NK cell biology (24). GCase activity in KHYG1 and target K562 cells was inhibited by adding CBE to a final concentration of 100 µM. Significant GCase inhibition was found in both CBE-treated cell lines (**Figure 2D**). As with CTL cells, 93% of GCase inhibition was achieved compared to control, but there was no significant effect on KHYG1 cell cytotoxicity (**Figure 2E**).

Overall, our findings suggested that inhibition of glucocerebrosidase activity in the CTL and NK cells of GD patients does not impair the cytotoxic activity of these cells.

## Discussion

In this study, we investigated the effect of GCase dysfunction on the effector function of cytotoxic lymphocytes. We found that CTL and NK cells from GD type I patients, as well as cells subjected to irreversible pharmacological inhibition of GCase, exhibited no change in cytotoxicity.

Familial and acquired HLH, as well as GD can present with similar clinical features, including cytopenia, hepatosplenomegaly, elevated serum ferritin level, fever, and systemic inflammation (16). The immune changes in GD patients have been previously characterized, including alterations in T and NK cell frequencies, but functional analysis of these cells has not been conducted (25). Two earlier studies (17, 19) investigated NK cells derived from GD patients by assessing perforin expression and found it to be unaffected. However, normal perforin expression does not necessarily indicate intact cytotoxic function of CTL/NK cells. For example, normal perforin levels are also observed in patients with FHL caused by mutations in the *UNC13D*, *STX11*, and *STXBP2* genes (13). Moreover, we recently discovered that another sphingolipidosis, Niemann-Pick disease type C1, NPC1, leads to severely impaired CTL function due to the suppression of perforin activity despite normal expression levels; this was hypothesized to be due to abnormal accumulation of cholesterol and sphingomyelin in the cytotoxic granules that store perforin (20). It is also worth noting that partial loss of cytotoxic lymphocyte effector function can present with variable, atypical symptoms that often do not meet all the diagnostic criteria for FHL, and this can potentially obscure a link between FHL-like disease and GD (14). Collectively, these considerations have prompted us to investigate the effect of GCase deficiency on the function of CTL/NK cells.

Although HLH is only occasionally associated with GD, patients may still present with acute symptoms that fulfill at least some diagnostic criteria of FHL/HLH (15–19). From a therapeutic point of view, particularly for younger patients, it is critically important to understand the underlying cause of these symptoms. While hematopoietic stem cell transplantation is curative for FHL, it can be detrimental for patients with HLH symptoms who are presumed to have FHL but do not, as their disease may be brought on by causes other than primary CTL/NK cell dysfunction.

Our findings indicate that cytotoxic lymphocyte function remains intact in Gaucher disease patients, suggesting that HLH-like symptoms arise from alternative mechanisms. Therefore, stem cell transplantation - the only curative treatment for familial HLH due to impaired cytotoxic lymphocyte function - may do more harm than good in these cases, given its substantial risk of morbidity and mortality. This insight also has implications for the treatment of GD patients who develop B cell malignancies: since their cytotoxic lymphocyte function remains intact, these patients may be suitable candidates for immunotherapy, which relies on the effector function of cytotoxic lymphocytes. This would not be the case if their cytotoxic function was compromised due to GCase deficiency.

In conclusion, our data show that CTL/NK cell function is not affected by GCase deficiency, indicating no association between GD and FHL-like symptoms due to impaired cytotoxicity. Instead, the symptoms observed in some GD patients are more likely driven by increased pro-inflammatory cytokine release from Gaucher macrophages (16). Future research should therefore focus on pro-inflammatory immune cells, such as macrophages, CD4 T helper cells, and mast cells, to better elucidate the mechanisms underlying secondary HLH-like symptoms in patients with Gaucher disease.

## Data Availability

The original contributions presented in the study are included in the article/**Supplementary Material**. Further inquiries can be directed to the corresponding author.
