# Supplementary material for: Cytotoxic lymphocyte effector function is unaffected in patients with Gaucher disease
Source: Front Immunol. 2025 Oct 16;16:1680520. doi: 10.3389/fimmu.2025.1680520 (PMC12571723; doi:10.3389/fimmu.2025.1680520)
Supplement: Supplementary file 1 [file DataSheet1.pdf]

Supplementary Material

1 Supplementary Figures

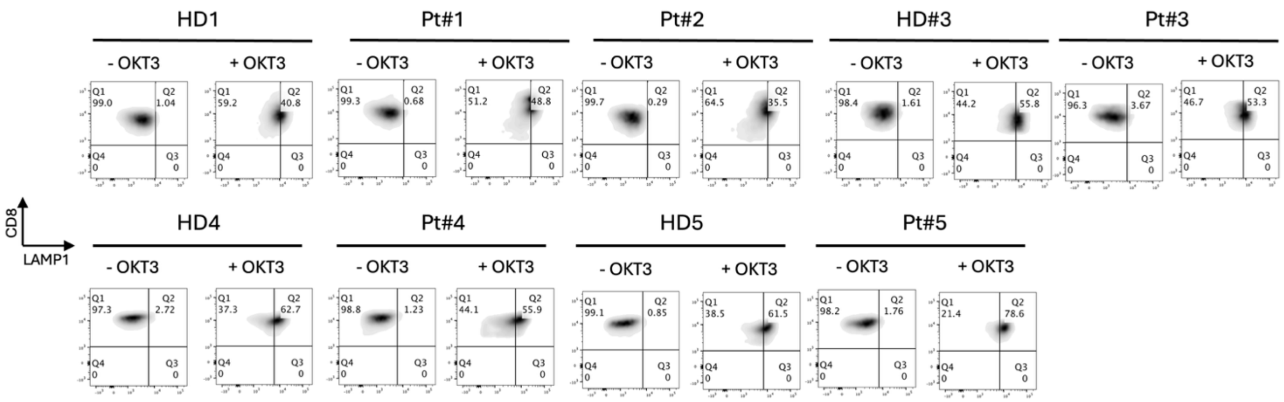

Supplementary Figure 1. Flow cytometry panels of patients' CTL degranulation. The ID numbers of a healthy donor correspond to the patient ID number processed on the same day. For example, HD#1 corresponds to Pt#1 and Pt#2, while HD#3 corresponds to Pt#3. In each panel, the population was selected for CD8+ cells. The LAMP1 cut-off gate was set based on the background LAMP1 level of each healthy donor in the absence of OKT3 and then applied to the patients' panels conducted on the same day. The percentage of extracellular LAMP1 was obtained by calculating the difference of the LAMP1-positive population between the absence (-OKT3) and the presence (+OKT3).

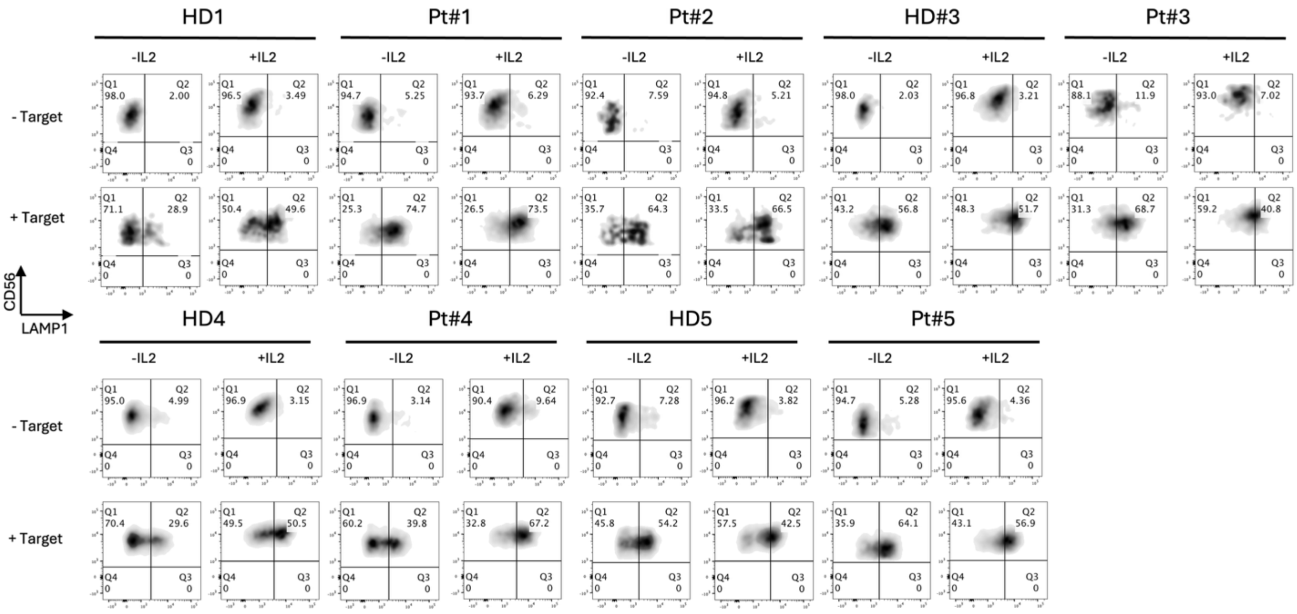

Supplementary Figure 2. Flow cytometry panels of NK degranulation of Patients #1 to #5 in the presence and absence of rhIL-2. NK cells derived from patients exhibited normal levels of degranulation in both the presence and absence of rhIL-2. The NK cell population was gated (CD3-, CD16+, CD56dim) from PBMCs. The percentage of extracellular LAMP1 is obtained by calculating the difference of the LAMP1-positive percentage between with and without target cells.

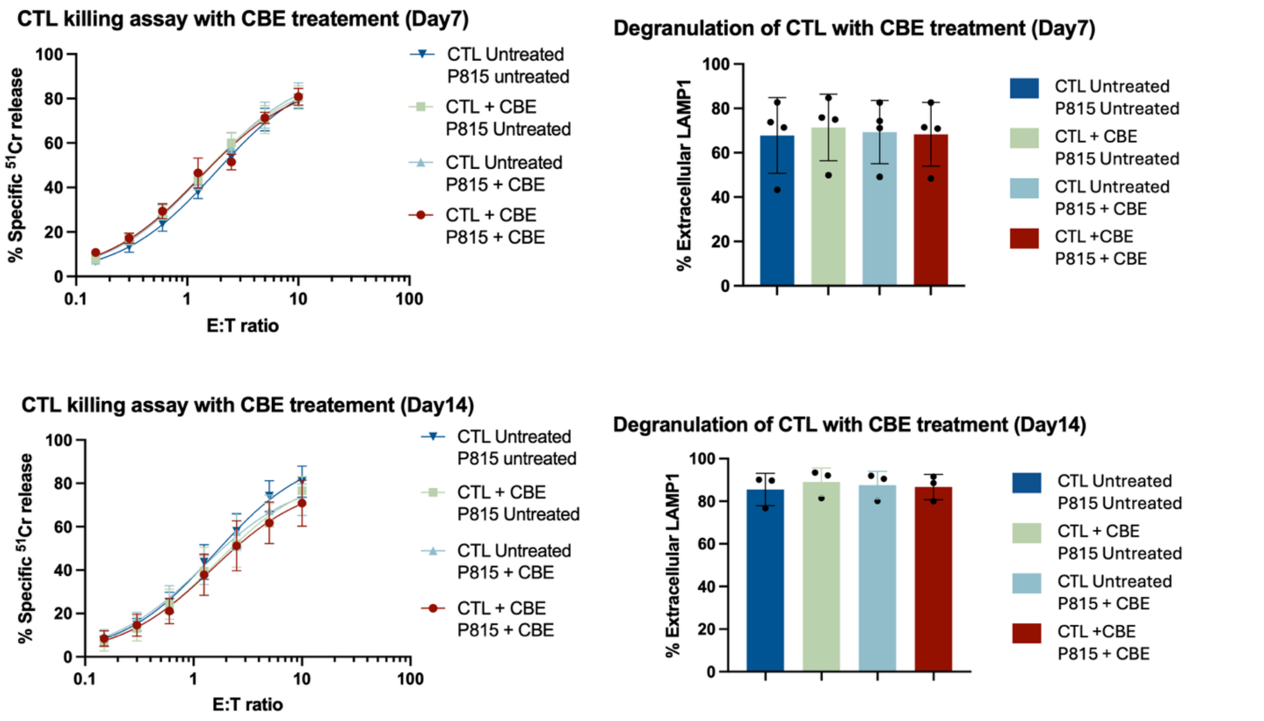

Supplementary Figure 3. The functional assays for CTLs and P815 treated with CBE. The functional assay was performed on day 7 and day 14 of CBE treatment. Single treatment of CBE on CTL or P815 did not affect cytotoxicity or degranulation of CTL. For day 7, n=4 for both killing and degranulation assays. For day 14, n=4 for the killing assay, and n=3 for the degranulation assay.

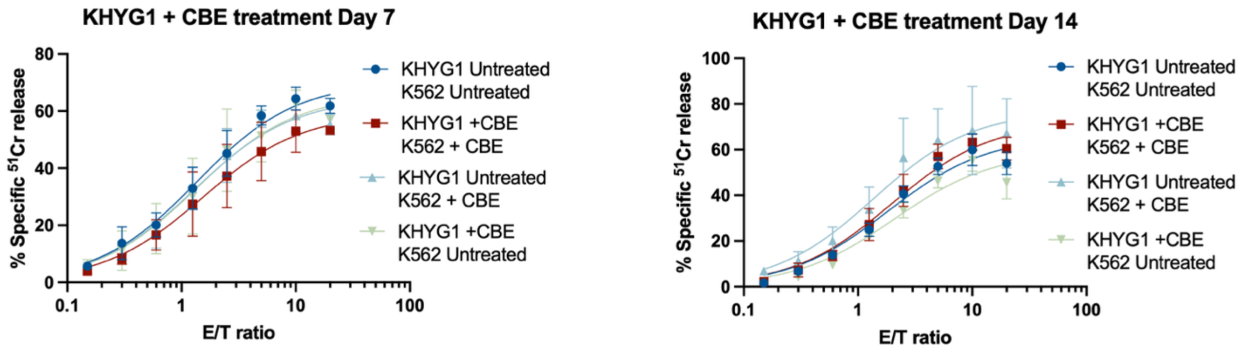

Supplementary Figure 4. The cytotoxic assays for KHYG1 and K562 treated with CBE. The functional assay was performed on day 7 and day 14 of CBE treatment. Single treatment of CBE on KHYG1 or K562 did not affect cytotoxicity. n=3 for both assays.
